# Evaluation of a novel ELISA for the tumor-associated antigen CA 72–4 in patients with ovarian cancer

**DOI:** 10.4155/fsoa-2016-0040

**Published:** 2016-10-12

**Authors:** Paul Buderath, Sabine Kasimir-Bauer, Bahriye Aktas, Jens Rasch, Rainer Kimmig, Thomas Zeller, Martin Heubner

**Affiliations:** 1 Department of Obstetrics & Gynecology, University Hospital Essen, Hufelandstr. 55, 45147 Essen, Germany; 2 DRG Instruments GmbH, Frauenbergstraße 18, 35039 Marburg, Germany

**Keywords:** CA 72–4, diagnostics, ELISA, ovarian cancer, tumor markers

## Abstract

**Aim:**

Cancer antigen 72–4 (CA 72–4) is an established tumor marker in ovarian cancer. We evaluated a new solid-phase ELISA (DRG TM-CA 72–4 ELISA).

**Materials & methods:**

Repeated measures of test samples and controls were performed to evaluate reliability and reproducibility. Afterward, we performed analyses on the sera of 150 patients with primarily diagnosed ovarian cancer. Results were compared with those of the Cobas CA 72–4 kit. Results were correlated with clinical patient data.

**Results:**

Results of the DRG TM-CA 72–4 ELISA were reproducible with acceptable deviations within measures, and the measured CA 72–4 serum concentrations were well in accordance with the references. High concentrations were significantly associated with grading, tumor stage and tumor residuals after surgery.

## Lay abstract

Cancer antigen 72–4 is a tumor marker for ovarian cancer. It can be measured in blood samples of patients to monitor the disease. We compared an established method of measurement with a newly designed one (DRG TM-CA 72–4 ELISA) and found that the newer one produced reliable results. Therefore, the DRG TM-CA 72–4 ELISA could be an option for the measurement of cancer antigen 72–4 in ovarian cancer patients.

**Figure 1.**
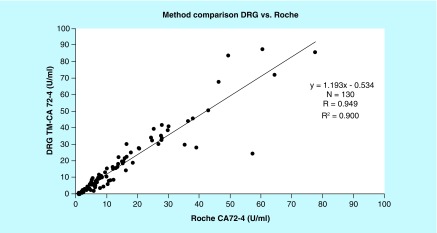
Correlation of the test methods revealed a correlation coefficient of 0.949.

**Figure 2.**
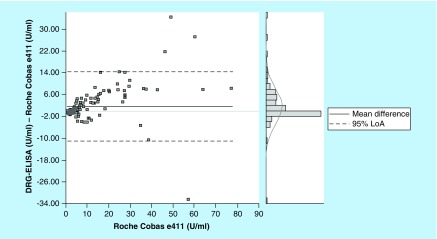
The Bland Altman blot illustrates the correlation in terms of deviations from mean and standard deviations.

Cancer antigen 72–4 (CA 72–4) was originally described as an antigenic determinant recognized by B72.3, a murine monoclonal antibody raised against a membrane extract of mammary carcinoma metastases [1]. Later, CA 72–4 was identified as a 1-MDa mucin-like glycoprotein complex termed tumor-associated antigen 72 (TAG-72) [2]. In 1989, Gero *et al.* developed a sequential assay using different monoclonal antibodies (B72.3 and CC-49) [3]. Since its discovery, TAG-72 has been detected on a broad variety of carcinoma cells. In their early works, Thor *et al.* found the glycoprotein to be present on more than 75% of the examined adenocarcinomas of different origin [4–6]. Especially, mucinous ovarian carcinomas showed high concentrations of TAG-72 [4]; other entities include gastric and endometrial cancer [7]. In a series of 110 patients with histologically proven primary ovarian cancer, Zeimet *et al.* found elevated concentrations of CA 72–4 in 42% [8]. This low sensitivity was contrasted by a high specificity of 99% in their collective. Schutter *et al.* evaluated a combination of physical examination, ultrasound, serum CA 125 and serum CA 72–4 in postmenopausal women with a pelvic mass, finding the highest specificity (93%) in CA 72–4 [9].

Since then, TAG-72 or CA 72–4 has been used as a marker for ovarian cancer both preoperatively and during follow-up. Today, CA 72–4, alongside CA 125, is a well-established marker used in the differential diagnosis of ovarian tumors as well as a valuable tool in follow-up of ovarian cancer patients [10,11]. In the study by Granato *et al.*, a combination of CA 72–4 with HE4 was shown to be superior to a combination of CA 125 with both CA 72–4 or HE4 in the detection of recurrent disease. In a 2016 retrospective study performed on blood specimen of 810 invasive epithelial ovarian cancer patients and 1939 healthy controls taken prior to the diagnosis of ovarian cancer, the combination of the best-established marker CA 125 with CA 72–4 led to a significant increase of specificity [12]. Furthermore, the marker has been used to differentiate between benign and malignant ovarian conditions. Anastasi *et al.* used CA 72–4 in the differential diagnosis between ovarian cancer and ovarian endometrioma [13].

CA 72–4 has also been analyzed regarding the evaluation of the extent of primary surgical tumor debulking. Fayed *et al.* described a good correlation of normal postoperative CA 72–4 levels with optimal cytoreductive surgery, thus showing a clear advantage of CA 72–4 over CA 125 in reflecting the residual tumor burden [14]. In the same series, CA 72–4 was elevated in 5/6 patients with overt clinical relapse compared with 4/6 for CA 125. This could be a further hint at an important role of the marker in the detection of relapse during follow-up.

In summary, CA 72–4 is a useful diagnostic tool especially in combination with other tumor markers such as CA 125 and HE4.

CA 72–4 is usually evaluated using a commercially available sandwich-ELISA assay using the aforementioned monoclonal antibodies B72.3 and CC-49. At our institution, measurement is performed using the Roche Diagnostics Cobas CA 72–4 kit on the Roche Cobas e411 (Roche Diagnostics, Mannheim, Germany). The aim of the present study was to validate the newly developed solid-phase DRG TM-CA 72–4 ELISA (DRG Instruments GmbH, Marburg, Germany). Therefore, we performed simultaneous measurements of CA 72–4 values in 150 serum samples of ovarian cancer patients, comparing the results between the two different assays.

## Material & methods

### DRG TM-CA 72–4 enzyme immunoassay

A total of 30 serum samples per day, 150 serum samples in total, of ovarian cancer patients were simultaneously measured with the DRG TM-CA 72–4 ELISA kit (DRG Instruments GmbH) on the Tecan Sunrise ELISA reader (Tecan Group AG, Maennedorf, Switzerland) and the Roche Diagnostics Cobas CA72–4 kit on the Roche Cobas e411 (Roche Diagnostics). The results of the Sunrise were calculated with the Tecan Magellan Software.

The DRG TM-CA 72–4 ELISA Kit is a solid-phase ELISA based on the sandwich principle. A unique antigenic site on a CA 72–4 molecule is captured by monoclonal mouse antibodies (clone CC-49) which were coated on the microtiter wells of the ELISA plate. After incubation of patient serum with enzyme conjugate, which is an anti-CA 72–4 antibody (clone B72.3) conjugated with horseradish peroxidase, the patients endogenous CA 72–4 is bound and the unbound conjugate was washed off. The amount of bound peroxidase is, after having added the substrate solution, proportional to the optical density (OD), exposed by the intensity of color developed and furthermore proportional to the concentration of CA 72–4 in the patient samples.

Serum matrix effects were evaluated in the assay validation with four different approaches as described in the test’s instructions for use [15]:

Parallelism of dilution could be demonstrated for three sera covering the complete measuring range Instructions for Use (Chapter 9.6);
Spike recovery was proven for three sera (see Chapter 9.5);
Hook effects could be excluded up to a concentration of 6400 U/ml.
No matrix effects could be observed by analyzing serum and plasma (EDTA, heparin and citrate).

All ELISA plates were prepared with five standards of different concentration. Using the Magellan software four-parameter Marquardt calculation, we generate a standard curve by applying standard OD against standard concentration. The patient samples’ concentration is calculated by using the patients OD on this standard curve.

All measures of CA 72–4 on Cobas e411 were performed in the Central Laboratory of the University Hospital Essen. Roche Cobas e411 uses the electrochemiluminescence (ECL) technology. ECL combines chemiluminescent analysis with electrode-controlled reactions, generating highly accurate results. Two labeled antibodies are added to the patient samples: first is labeled with ruthenium (Ru); second is labeled with biotin; both are specific to different epitopes of the endogenous CA 72–4 antigen, building a sandwich complex. In the next step, microbeats coated with streptavidin are added. The streptavidin binds strongly, noncovalently, on biotin. Now, the complex is transported into the measuring cell and a magnet is used to fix the complex with its microbeats under an optical unit. ‘ProCell’ solution is flushed into the measuring cell to wash out the unbound parts of the sample and to provide tripopylamine to the complex. The ECL reaction is triggered by voltage. Ru begins to emit a luminescent signal by losing an electron, regenerated by tripopylamine, which keeps the process running. The concentration of CA 72–4 is proportional to the ECL signal.

### Patients

A total of 150 patients with the first diagnosis of an ovarian malignancy who were treated in the University Hospital in Essen from 2004 to 2011 were included in the analysis. Controls consisted of healthy female voluntary blood donors. Informed consent was obtained from each patient. The median age of our patients was 59 years (ranging from 18 to 89 years); all patients were of Caucasian ethnicity. About 134 patients were suffered from epithelial ovarian cancer. For reasons of clarity, only these were included in further analyses concerning clinical associations. They were categorized into serous, mucinous and other tumors, the latter including endometrioid tumors and not further specified adenocarcinomas. Other, nonepithelial histopathological diagnoses not included in clinical data analyses comprised sex chord stromal tumors, carcinosarcomas and small-cell tumors. Detailed information on the patients’ clinical data is given in Table 1. Blood samples were drawn at the time of initial diagnosis before the patients underwent primary surgery and frozen afterward. Sample stability was proven after storage for 15 month at −20°C during assay verification. Though there is no final proof that the stability at −20°C can be extended to 10 years, CA–72.4 concentrations correlate well to tumor Fédération Internationale de Gynécologie et d'Obstétrique (FIGO) staging, grading and histopathology, indicating that there was no noteworthy degradation of 72.4 antigen during storage at −20°C.

The six test samples (negative, low positive [2×], moderate positive [2×] and one high positive) for reliability and reproducibility testing consisted of lyophilized human serum spiked with native CA 72–4 antigen. The two control samples were part of the DRG ELISA kit and were measured in each run to ensure the correctness of results.

To avoid systematic errors by diluting high-concentrated samples, all samples above the highest concentration of the DRG standard curve (S_max_ = 100 U/ml) were excluded from the method of comparison. The remaining 130 undiluted patient samples were used for evaluation.

### Statistics

Comparing different ELISA assays, a Bland–Altman analysis was conducted using the software Analyse-it for Microsoft Excel (version 3.70.1). For the analysis of potential associations with clinical data, the Kruskal–Wallis test was used (IBM SPSS Statistics, version 19).

## Results

In order to evaluate the reliability and precision of the DRG TM-CA 72–4 ELISA Kit according to the Clinical & Laboratory Standards Institute (CLSI) approved Guideline EP5-A (Evaluation of Precision Performance of Clinical Chemistry Devices), the test was established using a limited number of serum samples. Six test samples and two controls were analyzed in duplicates, two times a day (corresponding to two assay runs), on 20 days. The samples were measured on 20 nonconsecutive days, equivalent to a total of 80 measurements per sample. Analysis revealed an innerplate variation ranging from 3.0 to 5.1% and an interplate variation ranging from 5.8 to 10.3%. Detailed information is given in Table 2.

The dynamic range of the DRG TM-CA 72–4 ELISA is between LOQ = 0.82 U/ml and the highest concentration of the standard curve (S_max_ = 100 U/ml), whereby LOQ is defined as the CA 72–4 concentration that results in a coefficient of variation of 20% according to the CLSI-approved Guideline EP17-A (Protocols for Determination of Limits of Detection and Limits of Quantitation).

Comparing both measuring systems according to the CLSI-approved Guideline EP09-A2-IR (Method Comparison and Bias Estimation Using Patient Samples), the Bland–Altman test showed a good correlation of the test results with a correlation coefficient of 0.949 (Tables 3 & 4). The mean difference of results in between the assays was 1.61 U/ml with a standard error of 0.56 (0.49–2.72; 95% CI). Detailed information of the test results is depicted in Figures 1 & 2.

The analysis of associations with clinical data in patients suffering from epithelial ovarian malignancies showed that high concentrations of CA 72–4 were associated with tumor stage, histopathological grading and macroscopic tumor residuals after debulking surgery. Concerning grading and FIGO Stage, a linear association of the CA 72–4 concentration appears to apply showing lower concentrations in lower stages/grading and higher concentrations in advanced disease/high grading. Concerning the tumor resection status, the mean serum concentrations of CA 72–4 in not completely debulked patients is more than three-times higher than in patients who subsequently underwent macroscopically complete tumor resection. An overview on the results is given in Table 1.

## Discussion

Tumor markers including CA 72–4 can be helpful in the management of advanced ovarian cancer disease, especially in terms of monitoring during palliative therapy and if radiological examinations are inconclusive [10,11]. Concerning primary diagnosis, they might help discriminate between benign and malign tumors, although data show that the specificity is not satisfactory [9].

Our results show that the DRG TM-CA 72–4 ELISA is reliable and precise, and may be used in clinical practice. Compared with the well-established Cobas assay, it shows comparable and reproducible results. The small bias in the very low concentration range could rather be attributed to either calibration differences between both methods or differences in sensitivity (Roche Cobas and the DRG Elisa have detection limits of 0.2 and 0.79 U/ml, respectively).

From the viewpoint of efficacy, it is worth mentioning that less serum is needed for the DRG Assay compared to the Roche Cobas. Furthermore, it can be conducted by any unit which is familiar with immunoassays as it does not require special equipment. Hence, this ELISA might serve as an alternative diagnostic tool in clinical practice.

The association with clinical data reveals that high concentrations of CA 72–4 may correlate with negative prognostic factors such as tumor stage and grading. The fact that we found a correlation with postoperative tumor residuals although the blood samples had been drawn before surgery may account for the fact that there may be – as previously described by other authors [14] – a correlation of CA72–4 with large tumor burden, which again may lower the chances of achieving a macroscopically complete tumor resection. Although this finding does not directly impact upon the clinical routine in first-line therapy, it might be helpful in the situation of a platinum-sensitive relapse. In this situation, recurrent surgery is beneficial if a macroscopically complete tumor resection can be achieved. The CA 72–4 serum concentration might be of use in estimating the tumor burden in these patients. The observation that high concentrations of CA 72–4 are seen in advanced tumor stages also corresponds well to the reports of other authors [14]. Concerning the different histopathologic subtypes, the total numbers of patients with nonserous histopathology were too small to draw any statistic comparisons (see Table 1). However, there is no known relationship between histopathologic type of the primary tumor and sensitivity or specificity of the CA 72–4 assay.

Detailed analysis of six test samples and two controls in doublets on 20 nonconsecutive days showed that the results of the DRG TM-CA 72–4 ELISA were reproducible with acceptable deviations within measures. Additionally, we found that the measured CA 72–4 serum concentrations were well in accordance with the references.

Taken together, our data suggest that the DRG TM-CA 72–4 ELISA may be used in clinical practice in ovarian cancer patients as it shows comparable result to the frequently used Cobas assay.

## Conclusion

Taken together, our data suggest that the DRG TM-CA 72–4 ELISA may be used in clinical practice in ovarian cancer patients as it shows comparable results to the frequently used Cobas assay. The facts that the DRG essay needs less serum for the analysis and can be conducted by any unit familiar with immunoassays without any special equipment might make it an interesting alternative to the established Cobas essay.

## Future perspective

Given the affordability and feasibility of the DRG TM-CA 72–4 ELISA as well as the fact that the assay produces reliable and reproducible results, this new ELISA could represent a promising option for smaller laboratories wanting to measure CA 72–4 in patients’ serum. The efficacy of the test, needing smaller amounts of serum for analysis, could be of interest as well, especially against the background of the growing number of clinical and scientific parameters analyzed from the blood serum of oncologic patients.

**Table 1.** **Correlation of clinical data of 134 patients with epithelial ovarian cancer with results of the DRG TM-CA 72–4 ELISA assay.**

**Category**		**n**	**Mean CA 72–4 (U/ml)**	**Range (U/ml)**	**p-value according to the Kruskal–Wallis test**
FIGO tumor stage:					
– I		23	4.04	0–30.35	**<0.000**
– II		5	8.79	0–40.87	
– III		75	74.63	0–1001.53	
– IV		31	204.26	0–2090.88	
Grading:					
– 1		10	1.81	0–6.09	**0.003**
– 2		60	51.53	0–726.26	
– 3		64	139.94	0–2090.88	
Histopathology:					
– Serous		105	79.11	0–2090.88	**0.097**
– Mucinous		14	96.37	0–657.20	
– Other		15	160.69	0–1890.32	
Macroscopic tumor residuals after primary debulking:					
– Yes		61	152.26	0–2090.88	**0.001**
– No		73	38.07	0–635.15	

Bold indicates the statistically significant or close to statistically significant correlations.

CA 72–4: Cancer antigen 72–4; FIGO: Fédération Internationale de Gynécologie et d'Obstétrique.

**Table 2.** **Details on results of the DRG TM-CA 72–4 ELISA assay using 6 test samples and 2 controls (80 repeated measures per sample).**

**Sample**	**Mean CA 72–4 (U/ml)**	**SD (U/ml) within run**	**SD (U/ml) total**	**CV (%) within run**	**CV (%) total**
Sample 1	3.79	0.18	0.38	4.66	10.08
Sample 2	6.35	0.23	0.53	3.69	8.39
Sample 3	11.02	0.40	0.67	3.59	6.07
Sample 4	15.89	0.47	0.97	2.98	6.12
Sample 5	30.74	0.99	1.79	3.21	5.82
Sample 6	63.44	2.59	3.81	4.08	6.00
Control low	5.76	0.30	0.47	5.12	8.07
Control high	55.20	2.28	5.66	4.14	10.26

CA 72–4: Cancer antigen 72–4; SD: Standard deviation.

**Table 3.** **Details on the method comparison of Roche Cobas e411 and TM-CA 72–4 ELISA.**

**Method**	**Measuring interval**
Roche Cobas e411 (U/ml)	0.75–77.52
DRG-ELISA (U/ml)	0.00–87.45
Correlation coefficient	0.949

**Table 4.** **Details on the method comparison of Roche Cobas e411 and TM-CA 72-4 ELISA Part II.**

**Parameter**	**Estimate**	**95% CI**	**SE**
Mean difference	1.608	0.4920–2.7245	0.5642
95% lower LoA	−11.000	−12.9122–9.0871	0.9667
95% upper LoA	14.216	12.3036–16.1287	0.9667
SD	6.433		

LoA: Limits of agreement; SD: Standard deviation; SE: Standard error.

**Executive summary**

Cancer antigen 72–4(CA 72–4) is a useful diagnostic tool in the management of ovarian cancer, especially in combination with other tumor markers such as CA 125 and HE4.
CA 72–4 is usually evaluated using a commercially available sandwich-ELISA assay using the monoclonal antibodies B72.3 and CC-49.
The aim of the present study was to validate the newly developed solid-phase DRG TM-CA 72–4 ELISA (DRG Instruments GmbH, Marburg, Germany).
Therefore, we performed simultaneous measurements of CA 72–4 values in 150 serum samples of ovarian cancer patients, comparing the results between the two different assays.
A total of 150 serum samples of ovarian cancer patients were simultaneously measured with the DRG TM-CA 72–4 ELISA kit (DRG Instruments GmbH) and the Roche Diagnostics Cobas CA72–4 kit on the Roche Cobas e411 (Roche Diagnostics, Mannheim, Germany).
The DRG TM-CA 72–4 ELISA kit is a solid-phase ELISA based on the sandwich principle.
About 150 patients with the first diagnosis of an ovarian malignancy who were treated in the University Hospital in Essen from 2004 to 2011 were included in the analysis. Controls consisted of healthy female voluntary blood donors.
Comparing different ELISA assays, a Bland–Altman analysis was conducted. For the analysis of potential associations with clinical data, the Kruskal–Wallis test was used.
Comparing both measuring systems, the Bland–Altman test showed a good correlation of the test results with a correlation coefficient of 0.949.
The analysis of associations with clinical data showed that high concentrations of CA 72–4 were associated with tumor stage, histopathological grading and macroscopic tumor residuals after debulking surgery.
The mean serum concentrations of CA 72–4 in not completely debulked patients is more than three-times higher than in patients who underwent macroscopically complete tumor resection.
Our results show that the DRG TM-CA 72–4 ELISA is reliable and precise and may be used in clinical practice.
Compared with the well-established Cobas assay, it shows comparable and reproducible results.
Less serum is needed for the DRG assay and it can be conducted by any unit which is familiar with immunoassays as it does not require special equipment. Hence, this ELISA might serve as an alternative diagnostic tool in clinical practice.
The CA 72–4 serum concentration might be of use in identifying patients for recurrent surgery in the case of a platinum-sensitive relapse.

## References

[1] Colcher D, Hand PH, Nuti M, Schlom J (1981). A spectrum of monoclonal antibodies reactive with human mammary tumor cells. *Proc. Natl Acad. Sci. USA*.

[2] Johnson VG, Schlom J, Paterson AJ, Bennett J, Magnani JL, Colcher D (1986). Analysis of a human tumor-associated glycoprotein (TAG-72) identified by monoclonal antibody B72.3. *Cancer Res.*.

[3] Gero EJ, Colcher D, Ferroni P (1989). CA 72–4 radioimmunoassay for the detection of the TAG-72 carcinoma-associated antigen in serum of patients. *J. Clin. Lab. Anal.*.

[4] Thor A, Gorstein F, Ohuchi N, Szpak CA, Johnston WW, Schlom J (1986). Tumor-associated glycoprotein (TAG-72) in ovarian carcinomas defined by monoclonal antibody B72.3. *J. Natl Cancer Inst.*.

[5] Thor A, Ohuchi N, Szpak CA, Johnston WW, Schlom J (1986). Distribution of oncofetal antigen tumor-associated glycoprotein-72 defined by monoclonal antibody B72.3. *Cancer Res.*.

[6] Thor A, Viglione MJ, Muraro R, Ohuchi N, Schlom J, Gorstein F (1987). Monoclonal antibody B72.3 reactivity with human endometrium: a study of normal and malignant tissues. *Int. J. Gynecol. Pathol.*.

[7] Ohuchi NGE, Mort S, Akimoto M (1990). Clinical evaluation of CA 72 -4 immunoradiometric assay for serum TAG-72 antigen in patients with carcinoma. *J. Tumor Marker Oncol.*.

[8] Zeimet AG, Guadagni F, Marth C (1995). [Value of the TAG-72 (CA 72–4) tumor marker in primary diagnosis of ovarian carcinoma. A comparison with the established CA-125 marker]. *Geburtshilfe Frauenheilkd.*.

[9] Schutter EM, Sohn C, Kristen P (1998). Estimation of probability of malignancy using a logistic model combining physical examination, ultrasound, serum CA 125, and serum CA 72–4 in postmenopausal women with a pelvic mass: an international multicenter study. *Gynecol. Oncol.*.

[10] Granato T, Midulla C, Longo F, Colaprisca B, Frati L, Anastasi E (2012). Role of HE4, CA72.4, and CA125 in monitoring ovarian cancer. *Tumour Biol.*.

[11] Bian J, Li B, Kou XJ, Liu TZ, Ming L (2013). Clinical significance of combined detection of serum tumor markers in diagnosis of patients with ovarian cancer. *Asian Pac. J. Cancer Prev.*.

[12] Terry KL, Schock H, Fortner RT (2016). A prospective evaluation of early detection biomarkers for ovarian cancer in the European EPIC cohort. *Clin. Cancer Res.*.

[13] Anastasi E, Manganaro L, Granato T, Benedetti Panici P, Frati L, Porpora MG (2013). Is CA72–4 a useful biomarker in differential diagnosis between ovarian endometrioma and epithelial ovarian cancer?. *Dis. Mark.*.

[14] Fayed ST, Ahmad SM, Kassim SK, Khalifa A (1998). The value of CA 125 and CA72–4 in management of patients with epithelial ovarian cancer. *Dis. Mark.*.

[15] DRG Diagnostics http://www.drg-diagnostics.de/files/cla-4730_ifu--ca-72-4_2009-04-28_en_v1.0.pdf.

